# Reduction in Pulse Pressure during Standing Can Distinguish Neurogenic Orthostatic Hypotension

**DOI:** 10.3390/diagnostics11081331

**Published:** 2021-07-24

**Authors:** Kyu-On Jung, Deok-Hyun Heo, Eek-Sung Lee, Tae-Kyeong Lee

**Affiliations:** Department of Neurology, Soonchunhyang University College of Medicine, Bucheon 14584, Korea; laotheone@naver.com (K.-O.J.); yonyppappa@schmc.ac.kr (D.-H.H.); eeksung@schmc.ac.kr (E.-S.L.)

**Keywords:** neurogenic orthostatic hypotension, pulse pressure, head-up tilt test

## Abstract

Background: We investigated whether changes in the pulse pressure (PP) reduction ratio during the head-up tilt test (HUTT) can aid in distinguishing neurogenic orthostatic hypotension (OH) from non-neurogenic OH. Methods: We enrolled consecutive patients with NOH and non-neurogenic OH between January 2015 and October 2018. We compared the Valsalva ratio, the presence or absence of late phase II and IV overshoot, the pressure recovery time, and the PP reduction ratio during HUTT between the two OH groups. Results: The expiratory–inspiratory (E:I) ratio and Valsalva ratio were significantly decreased in the NOH group (*p* = 0.026, *p* < 0.001, respectively). The absence of late phase II and phase IV overshoot was more frequent in the NOH group than in the non-neurogenic OH group (*p* = 0.001, *p* < 0.001, respectively). The pressure recovery time was significantly prolonged in the NOH group (*p* < 0.001), which exhibited increases in the PP reduction ratio (1—minimal PP/baseline PP) during the HUTT (*p* < 0.001). We calculated the cutoff point for the PP reduction ratio during HUTT, which exhibited an area under the receiver operating characteristic curve of 0.766 (0.659–0.840, 95% confidence interval). The cutoff value for the PP reduction ratio during HUTT (0.571) exhibited sensitivity of 0.879 and specificity of 0.516. Conclusions: Increases in the PP reduction ratio during HUTT may be a meaningful NOH laboratory marker.

## 1. Introduction

Orthostatic hypotension (OH) can be classified into neurogenic OH (NOH) and non-neurogenic OH, according to its cause [1]. Differentiating these two entities is critical because diagnostic evaluations and treatment strategies are vastly different for NOH and non-neurogenic OH.

In the head-up tilt test (HUTT), NOH and non-neurogenic OH can be distinguished based on the presence or absence of compensatory increases in heart rate (HR) [1]. However, it can be difficult to distinguish the two in actual clinical practice, especially when the degree of HR increase is borderline. In addition, when neurogenic and non-neurogenic causes of OH coexist, non-neurogenic causes such as hypovolemia may mask NOH, showing a compensatory heart rate increase in clinically autonomic disorders.

Extensive autonomic function tests can be useful in such difficult cases. An absence of late phase II and phase IV overshoot accompanied by increases in pressure recovery time during the Valsalva maneuver generally suggests NOH [2,3]. However, it is sometimes impossible for patients to perform the Valsalva maneuver due to old age or underlying medical conditions. Therefore, it is necessary to develop a novel laboratory marker for NOH during the HUTT, as even patients in generally poor condition can perform this test.

In this study, we aimed to determine whether the pulse pressure (PP) reduction ratio during the HUTT is significantly higher in patients with NOH than in those with non-neurogenic OH. In addition, we evaluated whether progressive OH is more common among patients with NOH than among those with non-neurogenic OH.

## 2. Materials and Methods

### 2.1. Standard Protocol Approvals, Registrations, and Patient Consent

The present single-center, retrospective study was approved by the institutional review board of our medical center. The requirement for informed consent was waived.

### 2.2. Study Population

**Figure 1:**
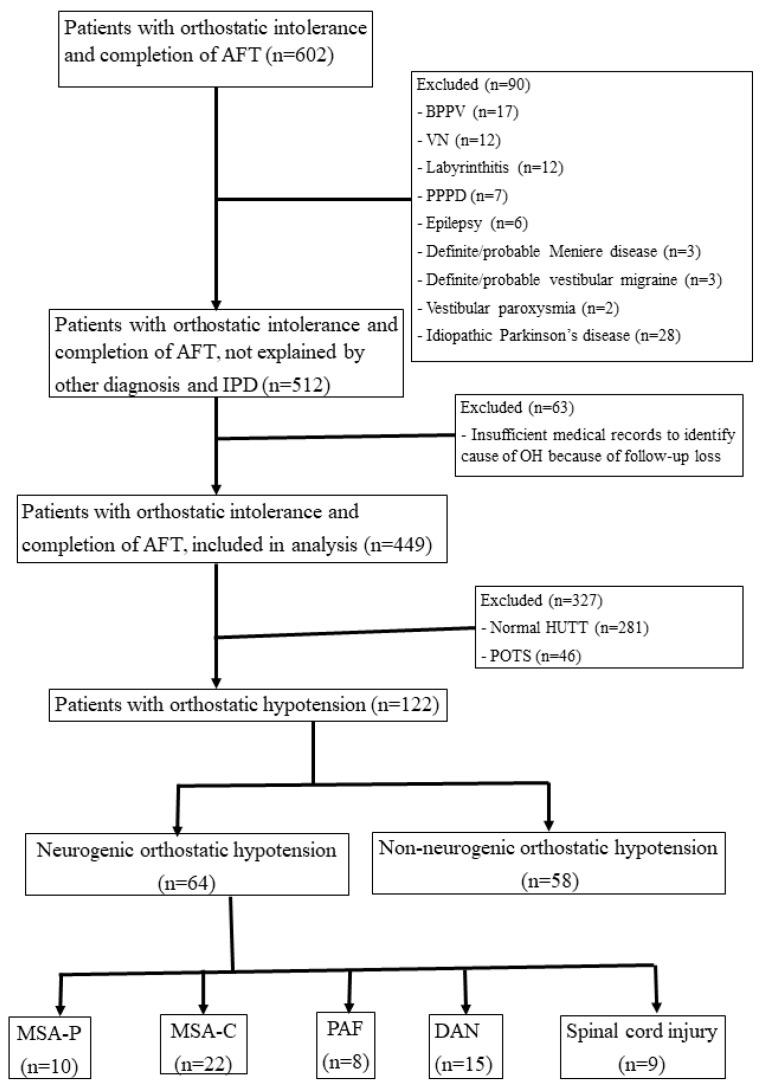
Flowchart for the inclusion and exclusion of patients for analysis. AFT: autonomic function test; IPD: idiopathic Parkinson’s disease; PPPD: persistent postural-perceptual dizziness; BPPV: benign paroxysmal positional vertigo; VN: vestibular neuritis; OH: orthostatic hypotension; HUTT: head-up tilt test; POTS: postural orthostatic tachycardia syndrome; MSA: multiple system atrophy; PAF: pure autonomic failure; DAN: diabetic autonomic neuropathy.

We included all patients who had undergone autonomic testing in the clinical autonomic laboratory of Soonchunhyang Bucheon Hospital between January 2015 and October 2018. The inclusion criteria were as follows: (1) a history of hemodynamic orthostatic dizziness/vertigo, (2) HUTT results fulfilling the consensus criteria for OH (Figure 1). Hemodynamic orthostatic dizziness/vertigo was defined in accordance with the diagnostic criteria [4]. OH was defined as a sustained reduction in systolic blood pressure (SBP) of at least 20 mmHg or diastolic blood pressure (DBP) of at least 10 mmHg within 3 min of standing, in accordance with consensus criteria [1]. In patients with hypertension and multiple system atrophy (MSA), OH was defined as a reduction in SBP of at least 30 mmHg or DBP of at least 15 mmHg within 3 min of standing [1]. The exclusion criteria were as follows: (1) insufficient medical records to identify the cause of OH due to loss of follow-up or other causes, (2) the presence of diseases that may be associated with orthostatic intolerance (benign paroxysmal positional vertigo, vestibular neuritis, vertebrobasilar insufficiency, persistent postural-perceptual dizziness, labyrinthitis, Meniere’s disease, vestibular migraine, vestibular paroxysmia, and epilepsy). Patients with vestibular neuritis, Meniere’s disease, persistent postural-perceptual dizziness, and vestibular migraine were diagnosed in accordance with the respective diagnostic criteria [5,6,7,8]. Patients with idiopathic Parkinson’s disease were also excluded because almost all were taking high doses of levodopa, and we could not differentiate OH due to the effects of levodopa.

All patient medical records were thoroughly reviewed for information regarding underlying causes of OH. In addition, we evaluated neurological findings; brain magnetic resonance imaging (MRI) and positron emission tomography-computed tomography (PET-CT) results (in some patients); and history of hypovolemia, heart failure, venous pooling, or use of medication known to cause OH. We also compared Composite Autonomic Symptom Score 31 (COMPASS 31) [9] results and orthostatic grades [10] between the two groups. This study was approved with a waiver of informed consent by the institutional review board of Soonchunhyang University Bucheon Hospital (IRB No. 2019-03-036).

### 2.3. Autonomic Testing

Patients underwent autonomic function tests based on established standards using the Finometer^®^ Pro system (Finapres Medical Systems, Amsterdam, The Netherlands) [11]. Patients performed deep breathing tests, the Valsalva maneuver, and the HUTT. Those who exhibited good performance status and were capable of the Valsalva maneuver performed both the Valsalva maneuver and the HUTT, while those incapable of the Valsalva maneuver performed the HUTT only. Medications that may have influenced autonomic test results could not be stopped because the sudden discontinuation of medications such as antihypertensive agents may harm patients.

### 2.4. Definition of NOH

Patients were classified into NOH and non-neurogenic OH groups based on autonomic function test results and clinical features. Inclusion criteria for the NOH group were as follows: (1) little or no compensatory increases in heart rate (less than 15 beats per minute) during the HUTT [1] and (2) a clinical diagnosis of central/peripheral autonomic neuropathy (e.g., possible and probable MSA, pure autonomic failure, diabetic autonomic neuropathy, and spinal cord injury). Diagnoses of probable and possible MSA were made based on the second consensus statement regarding MSA diagnosis [12]. Inclusion criteria for the non-neurogenic OH group were as follows: (1) compensatory increases in heart rate (over 15 beats per minute) during the HUTT and (2) a clear history of non-neurogenic causes, including hypovolemia or the use of antihypertensive agents, alpha-blockers, etc. Patients were required to meet all NOH criteria for inclusion in the NOH group.

### 2.5. Pulse Pressure Reduction Ratio and Definition of Progressive OH

We defined the PP reduction ratio as follows: 1—(minimal PP during the HUTT/baseline PP). Progressive OH was diagnosed when blood pressure decreased progressively throughout the HUTT [13].

### 2.6. Statistical Analysis

We compared autonomic test results and questionnaire responses between the NOH and non-neurogenic OH groups. For autonomic tests, we compared the expiratory–inspiratory (E:I) ratio, the Valsalva ratio, the presence or absence of late phase II and phase IV overshoot, the pressure recovery time, and the PP reduction ratio during the HUTT between the two groups. We also examined the frequency of progressive OH. In addition, we compared COMPASS31 results and orthostatic grades between the two groups. Independent two-sample t-tests were used to compare continuous variables, while Pearson chi-square tests or Fisher’s exact tests were used to compare categorical variables. Analysis of covariance (ANCOVA) was used to determine the PP reduction ratio, adjusting for demographic and clinical variables. The level of statistical significance was set as *p* value <0.05. Receiver operating characteristic (ROC) analysis was performed to determine the accuracy of the PP reduction ratio in predicting NOH. Youden’s method was used to determine an optimal cutoff point on the ROC curve for maximizing sensitivity and specificity [14].

### 2.7. Data Availability Statement

De-identified participant data are available upon reasonable request.

## 3. Results

**Figure 2:**
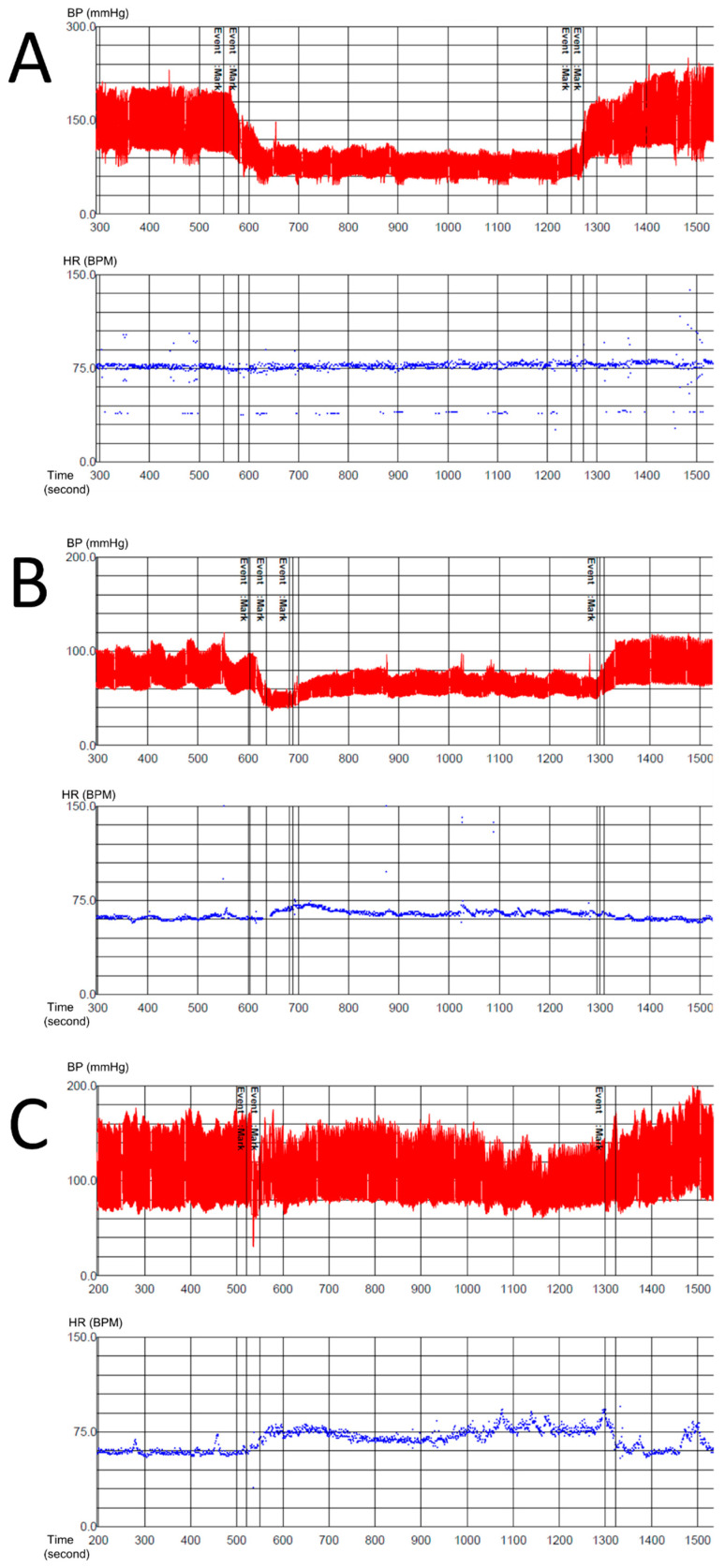
Representative examples of neurogenic OH (**A**,**B**) and non-neurogenic OH (**C**). A 63-year-old man clinically diagnosed with MSA-C exhibited typical NOH with high a PP reduction ratio (0.73) during the HUTT (**A**, vertical scale: 30 mmHg, horizontal scale: 100 s in upper row, vertical scale: 15 BPM, horizontal scale: 100 s in lower row). A 66-year-old woman clinically diagnosed with spinal cord injury exhibited typical NOH with a high PP reduction ratio (0.6) during the HUTT (**B**, vertical scale: 20 mmHg, horizontal scale: 100 s in upper row, vertical scale: 15 BPM, horizontal scale: 100 s in lower row). A 57-year-old man with hematemesis and anemia exhibited typical non-neurogenic OH with a low PP reduction ratio (0.4) during the HUTT (**C**, vertical scale: 20 mmHg, horizontal scale: 100 s in upper row, vertical scale: 15 BPM, horizontal scale: 100 s in lower row). BP: blood pressure; HR: heart rate; BPM: beats per minute; MSA-C: multiple system atrophy—cerebellum; OH: orthostatic hypotension; NOH: neurogenic orthostatic hypotension; PP: pulse pressure; HUTT: head-up tilt test.

We evaluated data for a total of 122 patients who fulfilled the inclusion criteria for OH, including 64 patients in the NOH group and 58 patients in the non-neurogenic OH group. Table 1 shows the characteristics of the included patients. Although the patients were gender-matched, they were not age-matched: patients in the NOH group were older than those in the non-neurogenic OH group because causative diseases of NOH such as MSA occur mostly in older adults [15]. Approximately 17% of NOH cases occur in patients over 65 years of age, while non-neurogenic OH can occur at any age [16]. Among all 122 patients, 13 could not perform the Valsalva maneuver properly and underwent the HUTT only. Among the 64 patients in the NOH group, 22 were diagnosed with MSA-C, 10 with MSA-p, 15 with diabetic autonomic neuropathy, 8 with PAF, and 9 with spinal cord injury. Among the 58 patients in the non-neurogenic OH group, 13 patients were related with drug side effects, 6 with hypovolemic states such as gastrointestinal bleeding, 1 with a heart problem, and the rest of them were undetermined. Figure 2 shows typical cases of NOH with a high PP reduction ratio and non-neurogenic OH with a low PP reduction ratio.

Table 2 and Table 3 show autonomic function test results for the included patients. The results for the Valsalva maneuver did not significantly differ from those reported in previous studies [2,3]. The E:I ratio was significantly lower in the NOH group than in the non-neurogenic OH group (*p* = 0.026, Table 2). The Valsalva ratio was also significantly lower in the NOH group (*p* < 0.001, Table 2). An absence of late phase II and phase IV overshoot was more likely to occur in the NOH group than in the non-neurogenic OH group (*p* = 0.001, *p* < 0.001, respectively, Table 2). The pressure recovery time was significantly prolonged in the NOH group (*p* < 0.001, Table 2).

**Figure 3:**
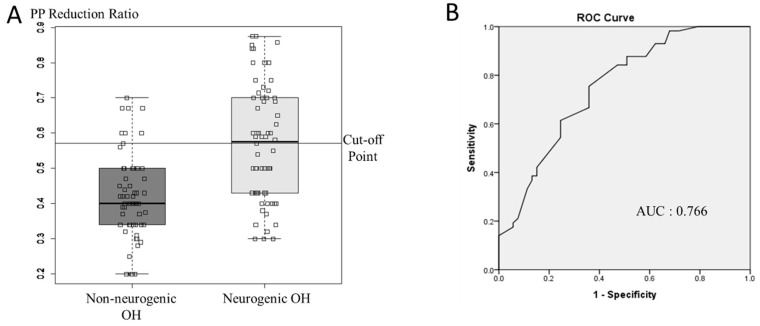
Pulse pressure reduction ratio (**A**) for each orthostatic hypotension subtype. Receiver operating characteristic (ROC) curve (**B**) for predicting high pulse pressure reduction ratio as a marker of neurogenic orthostatic hypotension. *p*: pulse pressure; OH: orthostatic hypotension; AUC: area under the curve.

The PP reduction ratio during the HUTT was significantly higher in the NOH group than in the non-neurogenic OH group after adjusting for covariates including age, DM, baseline SBP, baseline mean BP, mean BP change, and baseline PP. (*p* < 0.001, Table 3). The PP reduction ratio accurately predicted NOH, with an area under the receiver operating characteristic curve (AUC) of 0.766 (0.659–0.840, 95% confidence interval, Figure 3). The cutoff value for the PP reduction ratio (0.571) exhibited a good discriminatory ability for NOH and non-neurogenic OH, with a sensitivity of 0.879 and a specificity of 0.516. The predictive value for NOH was 0.755, while that for non-neurogenic OH was 0.738. Progressive OH was also more frequent in the NOH group than in the non-neurogenic OH group (*p* = 0.009).

Table 4 shows the questionnaire results for the included participants. Thirteen patients declined to complete the questionnaires. The total COMPASS31 was significantly higher in the NOH group than in the non-neurogenic OH group (*p* = 0.024, Table 4). Scores on the gastrointestinal and bladder domains were also significantly higher in the NOH group than in the non-neurogenic OH group (*p* = 0.007, *p* = 0.001, respectively, Table 4). There were no significant differences in the scores on other domains between the two groups. No significant differences in orthostatic grades were noted between the groups.

## 4. Discussion

In this study, we investigated whether changes in the PP reduction ratio during the HUTT can aid in distinguishing NOH from non-neurogenic OH. Indeed, our findings demonstrated that the PP reduction ratio during the HUTT could be used as a novel laboratory marker of NOH. Previous studies have verified that an absence of late phase II and phase IV overshoot accompanied by prolonged pressure recovery time can be used as laboratory markers of NOH [2,3]. Since some patients cannot successfully perform the Valsalva maneuver, these variables cannot be evaluated in some patients. However, the PP reduction ratio can easily be calculated for all patients with OH using HUTT results only, allowing for an accurate distinction between NOH and non-neurogenic OH. Additionally, even in patients with clinically diagnosed autonomic disorders, sometimes, HR increase occurs during the HUTT. Therefore, the PP reduction ratio can be a good novel laboratory marker for distinguishing NOH in these cases.

According to the Windkessel model, PP is a consequence of peripheral vascular compliance and stroke volume (SV) [16]. Compliance (C) is a measure of the capacity of the arterial system to compensate for increases in volume. PP is calculated by dividing SV by compliance (PP = SV/C) [16]. Thus, PP depends on left ventricular ejection and the properties of the arterial wall, which determine the compliance of the arterial system. These properties are affected by aging, atheroma, hypercholesterolemia, diabetes, and hypertension [16]. Normally, PP decreases during the HUTT as the venous return decreases due to venous pooling in the lower extremities [17]. However, if the baroreflexes fail, as in autonomic failure, PP may decrease beyond the normal range.

Previous reports have suggested that α1 adrenergic stimulation may lead to decreased vascular compliance [18] and increases in SV [19,20]. Thus, high PP reduction ratios in patients with NOH may be associated with decreased SV and increased vascular compliance due to adrenergic failure.

In accordance with previous findings, we also observed that an absence of late phase II/phase IV overshoot and prolonged recovery time were significantly associated with NOH [2,3]. Moreover, *p* values for decreased PP reduction ratios were even lower than those previously reported for autonomic findings in patients with NOH. COMPASS31 values were also significantly higher in the NOH group than in the non-neurogenic OH group, possibly because NOH is more likely to be associated with multi-domain autonomic failure [1]. As people with either NOH or non-neurogenic OH experience the same severity of symptoms related to orthostatic intolerance, we observed no significant differences in orthostatic grades between the two groups. 

Recent studies have aimed to identify novel laboratory markers to distinguish NOH from non-neurogenic OH based on HUTT results [21,22,23]. Kim et al. [22] suggested that if the magnitude of orthostatic SBP drop was greater than 36 mmHg and the magnitude of the orthostatic HR increase was smaller than 10 beats per minute at 1 min in the HUTT, the patient would likely be diagnosed with NOH. In addition, Norcliffe-Kaufmann suggested that a ΔHR/ΔSBP ratio < 0.5 during the HUTT can be diagnostic of NOH [23]. Although these markers can be useful, there are some limitations in certain clinical situations. For example, in patients with arrhythmia, including atrial fibrillation, which is common in the elderly and patients with anxiety disorders, these indicators cannot be applied because the heart rate may not be constant.

In this study, we also aimed to determine whether progressive OH occurs more frequently in patients with NOH than in those with non-neurogenic OH. Given that previous research has indicated that decreases in SBP are greater among patients with NOH than among those with non-neurogenic OH [22], we hypothesized that progressive OH might be another laboratory marker of NOH. We also observed that progressive OH was more prevalent in the NOH group than in the non-neurogenic OH group.

The present study has several limitations of note. First, possible factors that can affect compliance, including old age and diabetes, were more frequent in the NOH group than in the non-neurogenic OH group. Second, in consideration of patient safety, autonomic function tests were performed without stopping drugs that may lead to OH. Third, as we tried to include various causes of NOH, such as spinal cord injury as well as MSA, the study subjects were not homogeneous. Fourth, since it is a retrospective study and some patients were excluded from the analysis due to insufficient medical records, caution is required in interpreting the results.

## 5. Conclusions

It is important to discriminate NOH from non-neurogenic OH because diagnostic and therapeutic strategies differ greatly based on the type OH. As some patients cannot successfully perform the Valsalva maneuver, it is necessary to identify novel laboratory markers of NOH that can be derived from HUTT results. In addition, the PP reduction ratio evaluation can be useful when the heart rate is not constant, such as in patients with arrhythmia. Our findings demonstrated that a PP reduction ratio > 0.571 during the HUTT might be diagnostic of NOH. As an early diagnosis of NOH with HUTT becomes possible, the patient may undergo further evaluations, such as brain MRI and subsequent treatment earlier.

## Figures and Tables

**Table 1:** Patient characteristics.

Characteristics	Neurogenic OH (*n* = 64)	Non-Neurogenic OH (*n* = 58)	*p*-Value
Age	60.77 ± 10.86	50.05 ± 18.898	< 0.001
Sex			
Male	37 (57.8)	31 (53.4)	0.628
Baseline systolic BP (mmHg)	141.45 ± 22.67	130.33 ± 18.419	0.004
Baseline diastolic BP (mmHg)	76.03 ± 10.965	72.85 ± 9.032	0.084
Baseline mean BP (mmHg)	108.74 ± 15.413	101.59 ± 13.051	0.007
Minimal systolic BP (mmHg)	90.38 ± 22.282	92.76 ± 16.706	0.509
Minimal diastolic BP (mmHg)	61.33 ± 14.955	59.57 ± 13.254	0.495
Minimal mean BP (mmHg)	75.85 ± 17.586	76.16 ± 14.473	0.915
Mean BP change (mmHg)	32.89 ± 18.209	25.42 ± 10.325	0.007
Vascular risk factors			
Hypertension	20 (31.3)	19 (32.8)	0.858
Diabetes	17 (26.6)	1 (1.7)	< 0.001
Hyperlipidemia	9 (14.1)	9 (15.5)	0.821
Medication			
Antihypertensive agent	21 (32.8)	16 (27.6)	0.531
Alpha blocker	7 (10.9)	7 (12.1)	0.845
Antidepressant	4 (6.3)	3 (5.2)	0.798

Independent two-sample t-tests were used to compare continuous variables, while Pearson chi-square tests or Fisher’s exact tests were used to compare categorical variables. Continuous variables are expressed as the mean ± standard deviation, while categorical variables are expressed as *n* values with percentages. OH: orthostatic hypotension; BP: blood pressure.

**Table 2:** Valsalva maneuver results.

	Neurogenic OH (*n* = 53)	Non-Neurogenic OH (*n* = 56)	*p*-Value
E:I ratio			
Decreased E:I ratio	9 (17.0)	2 (3.6)	0.026
Valsalva ratio			
Decreased VR	44 (83.0)	18 (32.1)	<0.001
Late phase II			
Absent late phase II	35 (68.6)	19 (33.9)	0.001
Phase IV			
Absent phase IV	29 (56.9)	3 (5.4)	<0.001
Pressure recovery time	10.784 ± 12.984	2.477 ± 1.568	<0.001

Independent two-sample t-tests were used to compare continuous variables, while Pearson chi-square tests or Fisher’s exact tests were used to compare categorical variables. Continuous variables are expressed as the mean ± standard deviation, while categorical variables are expressed as *n* values with percentages. OH: orthostatic hypotension, E:I: expiratory–inspiratory, VR: Valsalva ratio; PP: pulse pressure.

**Table 3:** Head-up tilt test results.

	Neurogenic OH (*n* = 64)	Non-Neurogenic OH (*n* = 58)	*p*-Value
Baseline PP (mmHg)	65.42 ± 17.832	57.48 ± 12.657	0.006
Minimal PP (mmHg)	29.19 ± 14.309	33.19 ± 8.465	0.066
PP reduction ratio	0.57 ± 0.165	0.414 ± 0.123	<0.001
Type of OH			
Progressive OH	10 (15.6)	1 (1.7)	0.009

Independent two-sample t-tests were used to compare continuous variables, while Pearson chi-square tests or Fisher’s exact tests were used to compare categorical variables. Analysis of covariance (ANCOVA) was used to determine PP reduction ratio adjusting for covariates including age, DM, baseline SBP, baseline mean BP, mean BP change, and baseline PP. Continuous variables are expressed as the mean ± standard deviation, while categorical variables are expressed as *n* values with percentages. OH: orthostatic hypotension; PP: pulse pressure.

**Table 4:** COMPASS31 results and orthostatic grades.

	Neurogenic OH (*n* = 52)	Non-Neurogenic OH (*n* = 53)	*p*-Value
Total COMPASS31	34.617 ± 19.19	27.13 ± 14.047	0.024
Orthostatic intolerance	17.74 ± 13.846	15.93 ± 10.122	0.444
Vasomotor	0.22 ± 0.879	0.36 ± 0.914	0.420
Secretomotor	6.25 ± 3.785	5.01 ± 4.343	0.119
Gastrointestinal	5.53 ± 4.11	3.63 ± 2.867	0.007
Bladder	3.37 ± 3.398	1.38 ± 2.261	0.001
Pupillomotor	1.12 ± 1.385	0.83 ± 0.901	0.197
Orthostatic grading system	6.6 ± 5.746	5.44 ± 3.616	0.219
Frequency	1.55 ± 1.435	1.42 ± 1.054	0.615
Severity	1.49 ± 1.235	1.44 ± 1.018	0.828
Conditions	1.42 ± 1.336	1.23 ± 1.148	0.451
ADL	1.11 ± 1.311	0.81 ± 0.887	0.166
Standing time	1.00 ± 1.330	0.64 ± 0.929	0.106

Independent two-sample t-tests were used to compare continuous variables, which are expressed as the mean ± standard deviation. COMPASS: Composite Autonomic Symptom Score; ADL: activities of daily living.

## Data Availability

The authors declare that the data of this research are available from the correspondence author on request.

## References

[1] Low P.A. (2015). Neurogenic orthostatic hypotension: Pathophysiology and diagnosis. Am. J. Manag. Care.

[2] Vogel E.R., Sandroni P., Low P.A. (2005). Blood pressure recovery from Valsalva maneuver in patients with autonomic failure. Neurology.

[3] Schrezenmaier C., Singer W., Swift N.M., Sletten D., Tanabe J., Low P.A. (2007). Adrenergic and vagal baroreflex sensitivity in autonomic failure. Arch. Neurol..

[4] Kim H.A., Bisdorff A., Bronstein A.M., Lempert T., Rossi-Izquierdo M., Staab J.P., Strupp M., Kim J.S. (2019). Hemodynamic orthostatic dizziness/vertigo: Diagnostic criteria. J. Vestib. Res..

[5] Jeong S.H., Kim H.J., Kim J.S. (2013). Vestibular neuritis. Semin. Neurol..

[6] Lopez-Escamez J.A., Carey J., Chung W.H., Goebel J.A., Magnusson M., Mandalà M., Newman-Toker D.E., Strupp M., Suzuki M., Trabalzini F. (2015). Diagnostic criteria for Menière’s disease. J. Vestib. Res..

[7] Lempert T., Olesen J., Furman J., Waterston J., Seemungal B., Carey J., Bisdorff A., Versino M., Evers S., Newman-Toker D. (2012). Vestibular migraine: Diagnostic criteria. J. Vestib. Res..

[8] Staab J.P., Eckhardt-Henn A., Horii A., Jacob R., Strupp M., Brandt T., Bronstein A. (2017). Diagnostic criteria for persistent postural-perceptual dizziness (PPPD): Consensus document of the committee for the Classification of Vestibular Disorders of the Bárány Society. J. Vestib. Res..

[9] Sletten D.M., Suarez G.A., Low P.A., Mandrekar J., Singer W. (2012). COMPASS 31: A refined and abbreviated Composite Autonomic Symptom Score. Mayo Clin. Proc..

[10] Schrezenmaier C., Gehrking J.A., Hines S.M., Low P.A., Benrud-Larson L.M., Sandroni P. (2005). Evaluation of orthostatic hypotension: Relationship of a new self-report instrument to laboratory-based measures. Mayor. Clin. Proc..

[11] Novak P. (2011). Quantitative autonomic testing. J. Vis. Exp..

[12] Gilman S., Wenning G.K., Low P.A., Brooks D.J., Mathias C.J., Trojanowski J.Q., Wood N.W., Colosimo C., Dürr A., Fowler C.J. (2008). Second consensus statement on the diagnosis of multiple system atrophy. Neurologym.

[13] Brignole M. (2006). Progressive orthostatic hypotension in the elderly. E-J. Cardiol. Pract..

[14] Youden W.J. (1950). Index for rating diagnostic tests. Cancer.

[15] Kim H.A., Lee H. (2018). Pitfalls in the Diagnosis of Vertigo. J. Korean Neurol. Assoc..

[16] Dart A.M., Kingwell B.A. (2001). Pulse pressure-a review of mechanisms and clinical relevance. J. Am. Coll. Cardiol..

[17] Low P.A., Tomalia V.A. (2015). Orthostatic Hypotension: Mechanisms, Causes, Management. J. Clin. Neurol..

[18] Thiele R.H., Nemergut E.C., Lynch C. (2011). The physiologic implications of isolated alpha(1) adrenergic stimulation. Anesth. Analg..

[19] Fukuta I. (1972). Hemodynamic effects of beta adrenergic receptor stimulant and blockade. Hemodynamic effects of isoproterenol after propranolol. Nagoya J. Med. Sci..

[20] Joyce W., Axelsson M., Wang T. (2017). Autoregulation of cardiac output is overcome by adrenergic stimulation in the anaconda heart. J. Exp. Biol..

[21] Cheshire W.P., Goldstein D.S. (2019). Autonomic uprising: The tilt table test in autonomic medicine. Clin. Auton. Res..

[22] Kim H.A., Low P., Sletten D., Suarez M., Sandroni P., Fealey R., Coon E., Singer W. (2017). Neurogenic Versus Non-neurogenic Orthostatic Hypotension–Practical Predictors for the Office (P5. 323).

[23] Norcliffe-Kaufmann L., Kaufmann H., Palma J.A., Shibao C.A., Biaggioni I., Peltier A.C., Singer W., Low P.A., Goldstein D.S., Gibbons C.H. (2018). Orthostatic heart rate changes in patients with autonomic failure caused by neurodegenerative synucleinopathies. Ann. Neurol..

